# The Adverse Impact of Maternal Obesity on Intrapartum and Perinatal Outcomes

**DOI:** 10.5402/2012/939762

**Published:** 2012-12-20

**Authors:** Dimuthu Vinayagam, Edwin Chandraharan

**Affiliations:** Women's Directorate, St George's Healthcare NHS Trust, Blackshaw Road, London SW17 0QT, UK

## Abstract

*Background*. Confidential enquiries into maternal deaths in the UK have repeatedly highlighted increased maternal morbidity and mortality associated with maternal obesity. *Objective*. To determine the impact of increased body mass index (BMI) on intrapartum outcomes. *Materials and Methods*. A retrospective case-control analysis of intrapartum outcomes of the study group (100 women), with a BMI above 40 kg/m^2^ (class III Obesity) at booking and a control group (100 women) with a booking BMI between 20 and 25 kg/m^2^ was performed. *Results*. A statistically significant increase in delivery by caesarean section (OR 2.32, 95% CI 1.26–4.29), minor and major postpartum haemorrhage (OR 5.93, 95% CI 2.34–11.98, OR 16.11, 95% CI 2.08–125.09, resp.), perineal trauma (OR 2.59, 95% CI 1.44–4.69), and fetal macrosomia (OR 3.11, 95% CI 1.25–7.79) was noted in the study group. Babies also had an increased risk of having a lower APGAR scores in the study group as compared to the control group (OR 3.09, 95% CI 1.07–8.94). *Conclusion*. Women with a BMI > 40 kg/m^2^ experience increased incidence of intrapartum complications and hence, input of skilled birth attendants during labour is essential to improve intrapartum outcomes.

## 1. Introduction

There has been a staggering rise in the prevalence of obesity, both in the UK as well as worldwide. Obesity is a modern-day epidemic with implications across the whole of the healthcare services, not just maternity services. The worldwide prevalence has doubled in the past 30 years, with approximately 300 million women aged above 20 being classified as overweight [1]. Body Mass Index (BMI) is used as a universal method of classifying obesity. It is a simple index, calculated by dividing an individuals' weight in kilograms by the square of their height in metres squared.

UK prevalence rates for obesity in 2002 for females aged 16–24 and 25–34 was 11.3% and 20.9%, respectively [2]. In 2007, 24% of women aged above 16 yrs were classified as obese [3], indicating a significant rise. In 2010, 43 million children aged under 5 were classified as overweight [1] (BMI above 25) which has implications for the forthcoming generation of mothers.

The predominant aetiology of obesity is an imbalance between the amount of calories consumed and amount expended. The sharp rise in the prevalence of obesity is multifactorial, and amongst others, the result of changes in dietary habits, sedentary lifestyles, and urbanisation. The health implications of obesity are severe; there is a clear and well-documented association between obesity and cardiovascular disease, diabetes, and some cancers (e.g., endometrial).

A government report on obesity estimated the economic cost of obesity to the country at *£*3.3–*£*3.7 billion per year. At a local level, the impact on local maternity care providers must be taken into consideration as women with obesity may require additional resources including trained staff and appropriate bariatric equipment. 

The prevalence of maternal obesity is rising to become a modern day epidemic. In 2001, a study of 287,213 women in London reported a BMI greater than 30 kg/m^2^ in 10.9% of the cohort [4]. A more recent study looking particularly at the increase in incidence rates of maternal obesity over a 15-year period concluded that the incidence of maternal obesity had risen from 9.9% in 1990 to 16% in 2004. The study demonstrated a clear increase in the prevalence rate of obesity, furthermore they reported a predicted incidence of 22% in the year 2010, which they extrapolated from the trend they observed [5]. A national cohort study, using data obtained through the UK Obstetric surveillance system showed that the prevalence of extreme obesity (classified as a BMI above 50 kg/m^2^) was 9.3 per 10,000 [6]. Obese women were found to be older, caucasian, multiparous, and from routine and manual social groups.

The increase in the number of obese women requires healthcare providers to understand the implications and consequences of an increased BMI to both the pregnant women and her fetus. The most recent confidential enquiries into maternal deaths reported a total number of 261 deaths in the 2006–2008 triennium. The enquiry found that 30% of mothers who died from a direct cause and 24% of mothers dying from an indirect cause had a booking BMI above 30 kg/m^2^. 27% of deaths overall were in mothers classified as obese. The impact of maternal BMI on cause of death was most significant for thromboembolism, where 78% of deaths occurred in women with a booking BMI above 25 kg/m^2^. Cardiac disease, as in previous reports, was the leading cause of indirect maternal deaths and 61% of these cardiac deaths occurred in women classified as overweight or obese.

The adverse effects of obesity on pregnancy are widespread and well documented throughout the scientific literature. Apart from the increased rate of maternal and neonatal morbidity and mortality, there is also an increased risk of pregnancy loss, congenital abnormalities, gestational hypertension, and gestational diabetes, with the associated risk of iatrogenic premature delivery. Intrapartum risks include increased rates of emergency caesarean section, labour dystocia, and postpartum haemorrhage. Postpartum complications that appear to be higher in this group include infection, thromboembolism as well as prolonged hospital stay, and/or hospital readmission. Although a multitude of data exists that shows the increase in complications in this high risk group, there is sufficiently less information regarding the causes for these. Our traditional teaching of labour and its mechanical factors, consist of an understanding of the powers, the passenger, and the passage. We aim to look at how obesity affects these factors individually.

### 1.1. The Powers

Obesity has been linked to increased rates of caesarean sections. Although, a proportion of these will be for associated obstetric indications (e.g., failed IOL for gestational diabetes/hypertension), there is data to suggest that this is not completely explained by the high-risk obesity associated conditions alone [7]. High caesarean section rates have been documented in otherwise low-risk obese women, and it is therefore plausible that obesity in itself is an independent risk factor for labour dystocia, resulting in increased caesarean section [7, 8]. One study analysing 3913 pregnant women found that obese women, whether labouring spontaneously or induced, had a higher rate of caesarean section for arrest in the first stage of labour [7]. This finding, which has been reproduced in other studies [8] may be the result of poor uterine activity. Although, there is clearly some confounding due to difference in birth weight and other factors such as age, it has been shown that a greater number of first stage arrests occur in obese women than in normal-weight controls [7, 8].

Obesity in itself appears to have an adverse effect on uterine contractility, independent of macrosomia, gestation, or maternal age. Research into the biological and physiological basis that underlies this theory is growing.

There is an association with pregnancy and hypercholesterolaemia, which is further increased in obese patients. Cholesterol, which is present in cell membranes, has also been shown to play a role in smooth muscle contraction [9]. In vitro studies of myometrium obtained at elective caesarean section confirmed that strength and rate of contraction of myometrium is lower in obese than in normal-weight controls [7]. This could be the result of altered cholesterol levels which adversely affected the ability of the myometrial cells to contract. Dyslipidaemias result in changes in membrane viscosity and fluidity, which in turn affects the calcium ion influx during the contraction-relaxation of smooth muscle, thereby having a negative effect on contractility. Leptin is an adipose-derived hormone which has a role in metabolism and appetite stimulation. Leptin concentrations are known to be increased in obese individuals. In vitro studies have demonstrated a reduction in myometrial contractility caused by leptin [10].

Obesity may therefore be associated with dysfunctional uterine activity secondary to metabolic factors. Perhaps there are more metabolic factors which are yet to be determined.

### 1.2. The Passenger

Fetal macrosomia is a retrospective diagnosis made following delivery of a neonate weighing more than 4000 grams. Maternal obesity is associated with abnormal fetal growth. There appears to be a directly proportional relationship between maternal obesity and fetal macrosomia. In a meta-analysis [11], the prevalence rates of fetal macrosomia were 13.3% and 14.6% for obese and morbidly obese women, respectively, compared with 8.3% for the normal weight control group.

Fetal macrosomia in obese women is associated with an increase in birth weight of the fetus, as well as a change in its body composition. Sewell et al. [12] reported that the average fat mass of infants that were born to normal weight controls was 334 grams, whereas the infants of women with a BMI > 25 kg/m^2^ had a mean fat mass of 416 grams. This difference equates to a change in body fat composition of 11.6%. Clearly this observed increase in birth weight and macrosomic fetus' in this group can result in cephalopelvic disproportion resulting in the observed increase in caesarean section rate.

### 1.3. The Passage

One potential explanation of the effect of obesity, affecting the passage of labour, is the deposition of fat and increased adipose tissue within the maternal pelvis and birth canal. Crane et al. [13] postulated that the increased caesarean section rate that they observed in their study of 20,130 women, comparing obese with normal weight controls, may be related to increased deposition of soft tissue within the maternal pelvis leading to the observed increase in the caesarean section rate. One review carried out by Vahratian et al. [14] also reported an increased risk of unplanned caesarean section in the group they studied, however this was not as high as previously reported. The concept of soft tissue dystocia has not been directly supported by any evidence. Studies using medical imaging to quantify the fat deposition within the pelvis and correlate this with labour dystocia are lacking, and more evidence in this region needs to be sought.

## 2. Case-Control Analysis

We performed a retrospective case-control study of the last 100 women with a booking BMI greater than 40 (Class III Obesity), compared to a control group comprising of 100 normal BMI women (BMI 20–25 kg/m^2^). The BMI was calculated at the initial booking appointment, the first contact with antenatal services. It was therefore calculated using an early pregnancy bodyweight. We found that using the BMI at booking was the most consistent record for the subjects of BMI/weight at any point during the pregnancy. Our delivery unit is a tertiary referral centre, with over 5000 deliveries per annum.

Inclusion criteria for the study group were as follows.

- Booking BMI above 40 kg/m^2^.
- Singleton pregnancies.
- Cephalic presentation (confirmed either by ultrasound or by physical examination).
- Complete obstetric documentation/records.
- Complete neonatal documentation/records.

Inclusion criteria for the control group were as follows.

- Booking BMI between 20 kg/m^2^ and 25 kg/m^2^.
- Singleton pregnancies.
- Cephalic presentation (confirmed either by ultrasound or by physical examination).
- Complete obstetric documentation/records.
- Complete neonatal documentation/records.

## 3. Methods

The 100 subjects making up the study group were the last 100 women with a BMI greater than 40 kg/m^2^ who delivered on our unit, delivering between November 2010 and December 2011. The control subjects were matched for age, parity, and ethnic origin. The control subjects also delivered between the same timeframe on our unit.

Data collected included basic demographics, details of parity, maternal age at delivery, maternal BMI and weight at booking, and mode of onset of labour. We also collected data regarding mode of delivery, perineal trauma, blood loss at delivery, gestation at delivery, birth weight data, APGAR scores at 1 and 5 minutes following birth, and neonatal unit admission rates. 

All ventouse deliveries were performed using a hand-held Kiwi omnicup. Forceps deliveries were performed using nonrotational forceps. All caesarean sections (emergency and elective) and operative vaginal deliveries were performed by obstetric registrars or consultants.

## 4. Results

10% of the study group comprised of women with a booking BMI over 50 kg/m^2^ (morbidly obese). The average BMI in the study group was 44.5 kg/m^2^, whilst the average BMI of the control group was 22.9 kg/m^2^. The study group consisted of 32% nulliparous women and 68% of multiparous women, with an identical distribution of nulliparous and multiparous women in the control group. The mean age of the study group was 32.1 (range 16–47), and the mean age of the control group was 32.5 (range 22–47). 40% of the study group and 40% of the control group had their ethnic origin recorded as Caucasian. The proportion of Afro-Carribean women was higher in the study group (28%) than in the control group (19%).

The caesarean section rate in the study group was 41%, which is significantly higher than the 23% caesarean section rate observed in the control group. Overall, the risk of having a caesarean section was increased in the study group (OR 2.32, 95% CI 1.26–4.29). 19% of the study group were delivered by emergency caesarean section versus 10% of the control group (OR 2.11, 95% CI 0.93–4.80). The incidence of elective caesarean section was also higher in the obese group; 22% versus 13% (OR 1.89, 95% CI 0.89–3.99). 63% of the normal BMI group achieved a spontaneous vaginal delivery versus 51% of the raised BMI group (OR 1.63, 95% CI 0.93–2.87). The incidence of operative vaginal delivery was higher in the control group, 14% compared to 8% in the study group (OR 1.87, 95% CI 0.87–4.68). Contrary to what we expected, forceps were used either as the primary or sequential instrument, in 7% of births in the control group versus 2% of births in the study group. There was no difference in ventouse delivery rates between the groups (6% versus 7%).

Of the women who delivered vaginally, 27% had an intact perineum in the study group, as compared to 49% of the women in the control group (OR 2.59, 95% CI 1.44–4.69). Episiotomy rates were similar between the two groups (18% versus 15%).

Of the women who delivered vaginally, the incidence of postpartum haemorrhage (>500 mls) in the study group was 47%, compared with 13% in the control group (OR 5.93, 95% CI 2.34–11.98), demonstrating a significant increased incidence of postpartum haemorrhage in the study cohort. The incidence of major PPH (>1000 mls) in the study group was 14% versus 1% in the control group (OR 16.11, 95% CI 2.08–125.09) also a significant increase. The mean blood loss at spontaneous vaginal delivery in the study group was 558 mls, higher than the mean of 282 mls in the control group. Mean blood loss at elective caesarean section was 521 mls in the study group and 424 mls in the control group; mean blood loss at emergency caesarean section was also increased in the study group at 635 mls versus 525 mls in the control group. 

Oxytocin augmentation was required during the first stage of labour in 30% of the study cohort versus 22% of the control cohort (OR 1.52, 95% CI 0.80–2.87).

Preterm delivery was defined as birth occurring prior to 37 completed weeks of gestation. The incidence of preterm delivery was 6% in the study group versus 1% in the control group (OR 6.31, 95% CI 0.75–53.48). The mean birth weight in the control group was 3232 grams (range 1780 g to 4770 g) versus 3542 grams (range 2320 g to 5332 g) in the study group. The incidence of fetal macrosomia was significantly increased, 7% in the control group versus 19% in the study group (OR 3.11, 95% CI 1.25–7.79). We observed a significant difference in the incidence of poor APGAR scores (APGAR score less than 7 at 5 minutes) in term babies between the two groups; 5% in the control group versus 14% in the study group (OR 3.09, 95% CI 1.07–8.94). We did not observe a significant difference in admission rates to the neonatal unit between the two groups (6% versus 7%). 

## 5. Discussion

The purpose of this study was to analyse and compare the intrapartum events and outcomes in two populations; a study group consisting of 100 subjects with a booking BMI greater than 40 kg/m^2^ and a control group containing 100 subjects with a BMI at booking within the normal range, matched for parity, age, and ethnic origin, all delivering on the same unit within the same time frame. 

Our study showed a significant increased incidence of delivery by caesarean section in the obese group. These findings are consistent with other trials. Crane et al. [13] studied over 20,000 subjects, and concluded that increased prepregnancy weight was associated with an increased risk of delivery by caesarean section. More recently, Lynch et al. [15] studied over 5000 subjects in a retrospective cohort study, and showed that delivery by caesarean section was two- to threefold more likely in obese women. The same study also found that there was a progressive reduction in the successful vaginal delivery rate with increasing BMI, consistent with findings in our study. Arrowsmith et al. [16] carried out a large retrospective cohort analysis of nearly 30,000 women who were being induced for postmaturity. The authors found that a significantly higher proportion of obese women being induced ended up having a caesarean section when compared with normal weight controls.

Our study demonstrated an increased incidence of primary postpartum haemorrhage in the obese population. These findings have also been reported in other studies with larger cohorts [17]. Similarly there have been studies of large cohorts which have not shown an increased incidence of postpartum haemorrhage [16]. It is imperative to remember that blood loss documented at the time of delivery is an estimation, therefore a subjective value that is open to bias. However, we did observe a significant difference, although without laboratory comparisons, we would hesitate to make any conclusions regarding blood loss in obese women based on our findings. One possible mechanism that may increase the risk of bleeding is the previously discussed malfunction in uterine contractility secondary to increased cholesterol and leptin.

In our study, 30% of the study population required oxytocin augmentation versus 22% in the study group. Although we failed to show a statistically significant difference, we feel our finding is consistent with already published data. Vahratian et al. [14] found that the progress of labour was significantly slower with increasing BMI, in the 600 subjects that they studied. The biological basis of reduced powers in obese women has been described above. Whether augmentation with oxytocin is sufficient to overcome the diminished contractility, and therefore address the observed inadequate powers caused by obesity, is currently unknown.

Our analysis showed an incidence of fetal macrosomia (>4000 grams) of 19% in the babies born to obese mothers. This is consistent with reports already published [11]. Intrapartum complications of shoulder dystocia, nerve injury, clavicular fractures, reduced APGAR scores, and birth asphyxia are all increased in cases of fetal macrosomia. In England, median birth weights range from 2950 grams at 37 weeks gestation to 3610 at 41 weeks [18]. We have demonstrated an increased average birth weight in neonates born to obese women as compared to normal weight controls, consistent with previously published data. 

Although our study did not demonstrate a difference in admission rates to the neonatal unit, we did show an increased incidence of poor APGAR scores for neonates born to obese mothers. Perhaps this increase can be explained by various biochemical and metabolic derangements that neonates born to obese mothers may be more at risk. These range from hypoglycaemia, hyperglycaemia, and hypothermia to hyperbilirubinaemia [18, 19].

## 6. Conclusion

Obstetricians need to be acutely aware that obese patients form a high-risk population with an increased incidence of caesarean section, postpartum haemorrhage, and perineal trauma. We have also demonstrated the adverse perinatal consequences in association with obesity. Adequate precautions as well as experienced obstetricians and paediatricians should be available during birth in obese women, as the risks of adverse intrapartum and perinatal complications are increased in these women.

## References

[1] World Health Organization (2011). Obesity and overweight. *WHO Factsheet*.

[2] Office for National Statistics (UK ONS) General household survey.

[3] Office for National Statistics Statistics on obesity, physical activity and diet.

[4] House of Commons Health Committee Obesity: third report of session 2003-2004.

[5] Sebire NJ, Jolly M, Harris JP (2001). Maternal obesity and pregnancy outcome: a study of 287,213 pregnancies in London. *International Journal of Obesity*.

[6] Heslehurst N, Ells LJ, Simpson H, Batterham A, Wilkinson J, Summerbell CD (2007). Trends in maternal obesity incidence rates, demographic predictors, and health inequalities in 36,821 women over a 15-year period. *An International Journal of Obstetrics and Gynaecology*.

[7] Zhang J, Bricker L, Wray S, Quenby S (2007). Poor uterine contractility in obese women. *An International Journal of Obstetrics and Gynaecology*.

[8] Verdiales M, Pacheco C, Cohen WR (2009). The effect of maternal obesity on the course of labor. *Journal of Perinatal Medicine*.

[9] Babiychuk EB, Smith RD, Burdyga T, Babiychuk VS, Wray S, Draeger A (2004). Membrane cholesterol regulates smooth muscle phasic contraction. *Journal of Membrane Biology*.

[10] Moynihan AT, Hehir MP, Glavey SV, Smith TJ, Morrison JJ (2006). Inhibitory effect of leptin on human uterine contractility in vitro. *American Journal of Obstetrics and Gynecology*.

[11] Chu SY, Kim SY, Lau C (2007). Maternal obesity and risk of stillbirth: a metaanalysis. *American Journal of Obstetrics and Gynecology*.

[12] Sewell MF, Huston-Presley L, Super DM, Catalano P (2006). Increased neonatal fat mass, not lean body mass, is associated with maternal obesity. *American Journal of Obstetrics and Gynecology*.

[13] Crane SS, Wojtowycz MA, Dye TD, Aubry RH, Artal R (1997). Association between pre-pregnancy obesity and the risk of cesarean delivery. *Obstetrics and Gynecology*.

[14] Vahratian A, Zhang J, Troendle JF, Savitz DA, Siega-Riz AM (2004). Maternal prepregnancy overweight and obesity and the pattern of labor progression in term nulliparous women. *Obstetrics and Gynecology*.

[15] Lynch CM, Sexton DJ, Hession M, Morrison JJ (2008). Obesity and mode of delivery in primigravid and multigravid women. *American Journal of Perinatology*.

[16] Arrowsmith S, Wray S, Quenby S (2011). Maternal obesity and labour complications following induction of labour in prolonged pregnancy. *An International Journal of Obstetrics and Gynaecology*.

[17] Usha Kiran TS, Hemmadi S, Bethel J, Evans J (2005). Outcome of pregnancy in a woman with an increased body mass index. *An International Journal of Obstetrics and Gynaecology*.

[18] Hospital Episode Statistics, NHS Information Centre Table 30: median birth weight (grams) of live born singleton and multiple deliveries by gestation.

[19] Henriksen T (2008). The macrosomic fetus: a challenge in current obstetrics. *Acta Obstetricia et Gynecologica Scandinavica*.

